# Women's interdependence after hysterectomy: a qualitative study based on Roy adaptation model

**DOI:** 10.1186/s12905-022-01615-2

**Published:** 2022-02-13

**Authors:** Fatemeh Goudarzi, Talat Khadivzadeh, Abbas Ebadi, Raheleh Babazadeh

**Affiliations:** 1 Student Research Committee, Mashhad University of Medical Sciences, Mashhad, Iran; 2 School of Nursing and Midwifery, Mashhad University of Medical Sciences, Mashhad, Iran; 3 Nursing and Midwifery Care Research Center, Mashhad University of Medical Sciences, Mashhad, Iran; 4 Department of Midwifery, School of Nursing and Midwifery, Mashhad University of Medical Sciences, Mashhad, Iran; 5 Behavioral Sciences Research Center, Life Style Institute, Baqiyatallah University of Medical Sciences, Tehran, Iran; 6 Nursing Faculty, Baqiyatallah University of Medical Sciences, Tehran, Iran

**Keywords:** Hysterectomy, Interdependence, Qualitative Study, Roy adaptation model

## Abstract

**Background:**

Hysterectomy is a difficult process that some women encounter that can affect their interdependence, but its impact on women’s Interdependence has received less attention. Therefore, this study aimed to explain women’s Interdependence after hysterectomy.

**Methods:**

This qualitative study was performed using a directed content analysis approach in Mashhad (Iran). Thirty women with a history of hysterectomy were included in the study by purposive sampling method. Data were collected from August 2018 to November 2019 using semi-structured interviews based on the interdependence mod of the Roy adaptation model until data saturation. Data analysis was performed using MAXQDA software and the deductive approach of Elo and Kingas (J Adv Nurs 62(1):107–115, 2008. 10.1111/j.1365-2648.2007.04569.x).

**Results:**

Data analysis led to the production of 537 initial codes from participants’ experiences. By merging and categorizing them, the theme of “increasing interdependence” emerged, which consists of 2 categories: “Evolution independence and interaction with important people in life” and “Reinforced support system”, that include six subcategories.

**Conclusion:**

After hysterectomy, women not only feel a strong need for support from family members, especially their husbands, they are also seeking support from health care providers and their colleagues. Before the hysterectomy, it is recommended that family members be consulted to ensure the emotional support and care of women after the hysterectomy. It can help the adaptation to hysterectomy.

## Background

Hysterectomy is one of the most frequent gynecological surgeries. Prevalence of hysterectomy in countries such as the United States 26.2%, Australia 22 percent, Ireland 22.2 percent, Taiwan 8.8 percent, and Singapore 7.5 percent [1]. Culturally, the relationship between the uterus and the sense of femininity and sexuality, in addition to its role in fertility, has made the uterus an important organ for women [2]. Hysterectomy can be a difficult process for women. Since the uterus is a very important part of the body, its removal has physical and emotional consequences and it may lead to severe psychological reactions in women [3–5]. A psychological complication of hysterectomy includes depression [6], Anxiety [7], and stress [8]. The risk of pelvic floor prolapse, urinary incontinence, and sexual dysfunction are some of the physical complications associated with hysterectomy [9]. In addition to concerns about the surgery itself, hysterectomy also leads women to worry about the postoperative experience. The reason for this concern is the importance of the uterus for women. Silva et al. [10] stated that the uterus is important to the performance of women's social roles. The loss of an organ related to femininity such as the uterus is associated with decreased adaptation [11].

Hysterectomy can have a significant impact on a woman's life and social interactions [12, 13]. Social relationships include interactions between different people and how they affect each other, and include personal relationships, social support, and sexual activity [14]. Social interactions are characterized by distinct forms of interdependence [15]. Social interactions and social relations have been identified as important predictors of health and well-being. They are two aspects of social relations [11]. Researchers believe that social relationships can affect health and Social support promotes mental and physical health among patients [11, 16–19]. Different aspects of women's relationships with family, friends, co-workers, and health care providers affect their coping strategies [20]. Wu et al. found that receiving support from family and friends affected and strengthened women's confidence in accepting hysterectomy. These individuals provided emotional and informational support to the patient [21].

Roy Adaptation Model (RAM) is one of the nursing models that is commonly used in nursing research. In the RAM, human beings are considered as a system of adaptation that interacts with the environment. According to the RAM, the result of the mechanisms used by individuals to cope with stimuli is reflected in their behaviors. Behavior in RAM refers to the internal and external actions and reactions of individuals to stimuli and is defined as a set of feelings, thoughts, and behaviors of individuals. In the RAM, behaviors are described as adaptive or non-adaptive behaviors. Roy defined four modes of adaptation, including physiological, self-concept, role function, and interdependence. The physiologic mode is focused on the structures of the body and how they function. The self-concept mode includes individuals' feelings and thoughts about themselves. The focus of this mode is on the psychological and spiritual aspects of the individual. Role function mode includes behaviors related to the individual's roles in the family or community. It also refers to the expectations that must be met by the individual in each of these roles. The interdependence mode refers to the social and relational integrity of the individual and was also focused on giving and receiving social support [22]. In accordance with the purpose of this study, the mode of interdependence has been considered for this study. Interdependence is the process by which people interact with each other and it focuses on the impact of the consequences of each person's behavior on themselves and others [23]. Interdependence can provide fundamental insight into an individual's adaptation to the social environment [15]. In different diseases, interdependence has been studied based on the RAM. In a phenomenological study based on RAM, the experiences of patients, their families and nurses working in the intensive care unit during the critical illness period were examined and the result showed that there is an interdependence between all of them [24]. Also, the experience of Turkish pregnant women with nausea and vomiting during pregnancy was assessed using RAM and the participants' experience showed that they were dissatisfied with their relationships with important people in their lives and with supportive resources and disruption of interaction with their social environment [25]. In another study, the experience of patients who underwent bariatric surgery under the guidance of RAM was examined and the results showed that body image surgery improves social life, personal relationships and performance of participants [26]. The adaptation experiences of Turkish patients who underwent liver transplant surgery were evaluated according to the RAM. The results showed that the relationship between participants 'families and patients' interactions with others improved [27]. There wasn't found a qualitative study that examined the field of women's interdependence after hysterectomy.

The effects of removal of the uterus as an organ that is closely related to the definition of the role of women in society are often overlooked. In medical treatments, the focus is on physical recovery and providing care, so the issue of the being-woman, reduced to the secondary plan. Given that women are almost half of the community population, so they should be an important part of society to codify to health policy [10]. Understanding the impact of hysterectomy on women’s social relationships and interdependence enables health care providers to help women to experience the adaptation process with less difficulty. Shanti et al. [28] found that there is a significant relationship between the family support and adaptation process and the quality of life of women who underwent a hysterectomy. Given that there are differences between cultures in the experience of relationships, qualitative studies can provide an in-depth insight into the experiences of women perspective. Therefore, this study aimed to discover women’s experiences of interdependency adaptation after hysterectomy with a qualitative approach using the RAM.

## Materials and methods

This study was conducted by using a qualitative approach and a deductive content analysis method between June 2018 to October 2019.The directed content analysis approach can be used when there is a previous theory or research on a phenomenon but it is incomplete or needs further explanation. The purpose of this approach is to validate or expand a theoretical framework, concept, or theory. In this approach, the existing theory or research which guides the research is helpful in designing the research question and can also guide the determination of the initial coding scheme or relationships between codes [29]. The interdependence mode of The RAM was used as a framework for exploring the experience of the hysterectomized women's Socio- Emotional Interdependence. In a directed content analysis approach, the classification of the key concepts from the initial codes is determined based on a theory or concept. The open-ended questions used to collect data were based on reference categories based on RAM [29].

This study was performed in two referral centers for hysterectomy affiliated to Mashhad University of Medical Sciences Iran. The study population consisted of all the women who have undergone hysterectomy. Inclusion criteria were: the ability to speak Persian, having a history of hysterectomy surgery during the 6 months to past 10 years, interest in participating in the study, no mental disorder or dementia, no use of sedatives and drugs. Exclusion criteria were unwillingness to continue participation in the study. Purposive sampling was used with the highest Variety in age, educational level, occupation, cause of hysterectomy, time after hysterectomy and Menopausal status before hysterectomy, number of children, marital status.

The participants were identified based on the hospitalization information file. Using a phone call, while explaining the purpose of the study, they were invited to participate in the study. Data were collected using semi-structured in-depth interviews. In the interviews, open-ended questions were asked based on the interdependence mode of the RAM: “Describe your relationship with others since the time of hysterectomy." To get deeper information, questions such as “Would you please explain more?” and “would you please make your point clearer?” were asked. The interviews were held based on the convenience of the participants in a quiet place. Eventually, the data was saturated after the participation of 30 partisans. The interviews lasted between 30 and 96 min (average 60 min). With the conscious written consent of the participant, the interviews were recorded and transcribed verbatim immediately after each interview.

Data analysis was performed using the deductive content analysis of Elo and Kingas [30]. With MAXQDA software (version 10, VERBI Software, Berlin, Germany). In this approach, the researcher begins coding and categorization by identifying key concepts or variables using existing theory or previous research [29]. In this study, the main concepts were identified based on the interdependence mode and the coding and categorization process began. At first, the transcribed version of the interviews was prepared by the first author and then read several times to get a general understanding of their content. The text of all interviews was selected as the meaning unit. An unconstrained matrix of analysis was developed according to the interdependence mode of the RAM. While reading the text, Sentences or paragraphs were reviewed and Phrases, sentences, and paragraphs related to the Purpose of the study were identified and coded. The codes were placed in the matrix of analysis based on their similarities to the concepts of the interdependence mode RAM matrix, and then the codes were grouped into subcategories according to similarities. Larger categories emerged from grouping subcategories based on similarities and differences of concepts. This process continued until the main theme emerged. The data analysis process was discussed during several joint meetings of the research team to reach a consensus.

For the credibility of the data, enough time was spent collecting and analyzing the data. The researcher was long time involved in the field of research and data. The research team reached a consensus regarding data analysis. The members of the research team were experts in qualitative studies and had experience working in medical and educational centers in the field of gynecology and midwifery. The accuracy of the interview text was confirmed by some participants after transcription. For the dependability of the data, the data analysis process was verified by two external evaluators familiar with qualitative study and gynecological diseases. To ensure confirmability, the processes of study and details of data analysis were recorded and reported. The quotations of the participants were presented according to their statements. For transferability, the demographic characteristics of the participants and the field of study were described in detail. With the entry of participants with different demographic characteristics to study and reporting the study process carefully, it is possible for the reader to decide on the use of the results.

## Results

In this study, Key concepts and categorization of initial codes were conducted and reported based on the predicted categorization in the interdependence mode of RAM. The demographic characteristics of the participants have been summarized in Table 1. Data analysis led to the product of 537 initial codes from the participants’ experiences, by merging the similar codes, 124 codes were obtained. Finally, the theme of increasing interdependence was emerged, which includes two categories and six sub-categories (Table 2).

**Table 1:** Sociodemographic characteristics of the participants

Child number	Menopause status	Marital status	Hysterectomy duration (year)	Hysterectomy reason	Job	Education level	Age	Participant code
2	Non-menopausal	Married	1	Fibroma	Housewife	High school	39	P1
2	Non-menopausal	Married	3	Fibroma	Retired	University	54	P2
2	Non-menopausal	Married	10	CIN2	Retired	University	57	P3
3	Non-menopausal	Married	1	Bleeding	Housewife	Junior School	44	P4
2	Non-menopausal	Married	3	Irregular bleeding	Teacher	University	43	P5
1	Non-menopausal	Divorced	4	Atypical endometrial hyperplasia	Nurse	University	36	P6
3	Non-menopausal	Married	2	Placenta accrete	Housewife	High school	35	P7
0	Menopause	Married	1	Atypical endometrial hyperplasia	Retired	University	54	P8
1	Non-menopausal	divorced	4	Cervical cancer	Secretary	University	46	P9
4	Non-menopausal	Married	4	Fibroma	Housewife	Junior School	46	P10
2	Non-menopausal	Married	4	Atypical endometrial hyperplasia	Housewife	Elementary	40	P11
4	Non-menopausal	Married	3	Endometrial cancer	Housewife	Junior School	48	P12
3	Non-menopausal	Married	1	Fibroma	Teacher	University	40	P13
3	Non-menopausal	Married	3	Fibroma	Housewife	Junior School	48	P14
3	Non-menopausal	Married	2	Malignant Fibroma	Housewife	High school	40	P15
3	Non-menopausal	Married	2	Ovarian cancer	Housewife	Elementary	43	P16
2	Non-menopausal	Married	8	Atypical endometrial hyperplasia	Retired	University	53	P17
3	Non-menopausal	Married	5	Placenta accrete	Housewife	Junior School	42	P18
0	Non-menopausal	Single	1	Fibroma	Employee	High school	37	P19
2	Non-menopausal	Married	2	Placenta accrete	Housewife	High school	37	P20
2	Non-menopausal	Married	3	Postpartum hemorrhage	Housewife	High school	33	P21
2	Non-menopausal	Married	1	Fibroma	Housewife	High school	45	P22
3	Menopause	Widow	1	CINIII	Housewife	Elementary	52	P23
2	Non-menopausal	Married	3	Fibroma	Housewife	Elementary	47	P24
5	Non-menopausal	Married	2	Fibroma	Housewife	Elementary	51	P25
3	Non-menopausal	Widow	1	Fibroma	Housewife	Elementary	45	P26
1	Non-menopausal	Married	2	Ovarian cancer	Housewife	University	40	P27
3	Non-menopausal	Married	3	Postpartum hemorrhage	Housewife	Elementary	27	P28
10	Menopause	Married	8	postmenopausal Bleeding	Housewife	Elementary	68	P29
1	Menopause	Married	9	Uterine prolapse	Housewife	Elementary	60	P30

**Table 2:** Main categories and subcategories of interdependence after hysterectomy

Cod	Sub-category	Category	Theme
Fading relationship with God	Evolution in spiritual relation	Evolution in dependence and interaction with important people in life	Increasing interdependence
Reducing communication with God			
Maintaining the relationship with God			
Improving communication with God			
Increasing dependence on offspring	Fluctuation in emotional dependency on offspring		
more intimacy with offspring			
Increasing relationship with offspring			
Increasing interest in offspring			
becoming more kind to offspring			
Expressing more love to offspring			
Taking Care of Kids with more sensitivity			
Hiding grief due to hysterectomy from offspring			
Trying to reduce offspring dependence			
Reducing attachment to the offspring			
No change in dependency to the offspring			
more intimacy to the spouse	Change in emotional relationship from the spouse		
Improving emotional attachment with spouse			
augmenting love and affection to the spouse			
The tendency to spend more time with spouse			
Need to be with the spouse			
being relaxed in the presence of the spouse			
The desire to have a close relationship with a spouse			
showing appreciation to the husband with Paying more attention to his support			
Strengthening friendship with husband			
Spend more time with spouse			
Improving emotional relationship with the spouse			
Compassion for the spouse due to limited sexual Activity			
Impaired emotional relationship with the spouse			
Not talking about self-issues with the spouse			
Not accompanying with spouse			
Feeling apathy to the spouse			
Tendency for independence from the spouse			
Receiving consolation from the spouse	The family as a refuge to receive emotional support and care	Reinforced support system	
the companionship of the spouse in a lonely time			
receiving spouse financial support			
receiving spouse care			
receiving care from family members			
receiving care from relatives			
Trusting to receive family support in difficulty			
Taking refuge to the family to escape anxiety			
Taking refuge to the family to escape anxiety			
Receiving Consolation from the family			
Prioritizing attending family gatherings rather than attending other gatherings			
Receiving encouraging and supporting colleagues to pursue treatment	Supportive friends and peers		
Increased cooperation of colleagues in performing my duties due to physical disability			
Increasing the emotional support received from others			
Increasing communication with hysterectomies women			
Receiving guidance from hysterectomies women			
Increasing emotional relationship with supportive friends			
Taking refuge in friends			
Spending more time with friends			
Reducing contact with friends to preventing stigma			
Cutting off relationship with some of friend			
The disappointment of friends due to lack of support			
Receiving support from the physician	Supportive health providers		
Receiving emotional support and respect from nurse			
Understanding affectionate communication of hospital staff			

### Evolution in dependence and interaction with important people in life

Most participants in this study reported experiencing a sense of loss after a hysterectomy, which affected their emotional state and emotional relationships. In the present study, participants experienced a change in their relationships with people who were important in their lives after hysterectomy. These changes ranged from increasing to decreasing relationships. This category consists of three subcategories.

#### Evolution in spiritual relation

Most participants experienced fear and unreliability after a hysterectomy. Most participants reported that they sought refuge in a source of power to overcome this fear and uncertainty. They often improved their spiritual relationship with God as absolute power. A participant stated that:

“My communication with God has been increased. I feel as if he was on my side during this difficult time. I talk to him more. I relied on God.” (P16)

A small number of participants decreased their relationship with God and complained to God about the loss of their uterus. One of the participants said:

“I had so many problems after the hysterectomy. I complained to God. I was ungrateful… I lost my relationship with God…” (P21)

#### Fluctuation in emotional dependency on offspring

Most participants reported losing their fertility after uterine surgery. Therefore, they became emotionally dependent on their children. They described knowing that current children are the only possible children for them to increase their dependence, intimacy with their children, and described their close relationship with their children. One of the participants said:

“I missed the opportunity to have children so my dependence on my children increased greatly. I had only these two children.” (P22)

A small group of participants described their negative psychological experiences after hysterectomy and said that these experiences reduced their dependence on their children. Participants described decreased attachment, efforts to reduce dependency, and reduced intimacy with children. A participant said:

“I have blame others, even my children in my hysterectomy. If I had not become pregnant, I would have remained a healthy and complete person. I am no longer as attached to my children as I used to be.” (P28)

For many participants in this study, hysterectomy was the main stimulus for dependence on children So they were more committed to caring for their children. Taking every opportunity to express love and affection for her children was a behavior that these women displayed due to the loss of their fertility. They said they were more sensitive to their children's future and health. One participant said:

“I can no longer have a child, so I have to take more care of my children so that nothing happens to them…” (P1)

#### Change in emotional relationship from the spouse

Most of the participants stated that hysterectomy affected their emotional relationship with their spouses. Most of the participants described the improvement in their emotional relationship with their spouses because of the support that they received from their spouses after the hysterectomy. Some said that being with their spouse can calm them down. Others indicated that the presence of their spouse during the illness and treatment enhanced their interest. A group also described the need to be with their spouse. Some also wanted to have a more intimate relationship with their spouse. One participant said:

“After the hysterectomy, I had a better relationship with my husband than before. I have peace by his side. We spend more time together.” (P14)

Some of participant stated that the loss of the uterus made women feel void and deficient. They were concerned about their spouses' reaction to their condition. This concern led some of these women to describe problems in their relationship with their husbands. They were reluctant to talk about their personal issues with their spouses. They did not accompany their spouses for work and leisure, they felt disinterested in their spouses, and some said they wanted to be independent of their spouses. One participant said:

“After the hysterectomy, I try to distance myself from him… I'm worried about what he thinks of me. I will not talk to him about myself anymore.” (p13)

Some women did not receive enough support from their husbands after hysterectomy, and as a result, they felt lonely and abandoned. Lack of support from the spouse harmed women's emotional relationships with their husbands and caused an emotional breakdown between them. One participant said:

“If I had not been emotionally attached to my husband, his absence after hysterectomy would not have upset me. I'm disappointed in him. I cannot communicate with him easily.” (P7)

Some participants owed themselves to their spouse's loving behavior. They tried to compensate for the loving behavior of their wives. They strengthened their emotional relationship with their spouse. They tried to improve their emotional relationship with their spouse. They stated that the relationship between them has become friendlier than in the past. One of the participants said:

“I am owing to my husband's behavior. He was committed to me during the difficult surgical conditions. Now I appreciate my husband more. I like him more.” (P15)

### Reinforced support system

In this study, a support system including family, colleagues, friends, and health care providers were described that supported participants in the areas of physical, care, emotional, and informational support. This category consists of three sub-categories.

#### The family as a refuge to receive emotional support and care

For most participants, the family served as a refuge for emotional support and care. Many participants said that their family members comforted them. Some women stated that after hysterectomy, they welcomed their husbands' supportive-caring behaviors. Some participants also described the spouses' financial support. A group of women described receiving family care, including their husbands, parents, sisters, and even relatives. They stated that when they felt lonely, they found their spouse by their side. Some participants stated that they sought refuge with their families when they experienced complications such as anxiety and stress after a hysterectomy. Being with family members calms them down. They trusted to receive support from their family in times of trouble. Although they were reluctant to attend public gatherings, they preferred to attend family gatherings. One participant said:

“I have been stressed and anxious since the hysterectomy. So I take refuge in my family (father, mother, and sisters). I am at peace with them. They comfort me.” (P28)

#### Supportive friends and peers

Many women after hysterectomy describe physical limitations, especially in the workplace and in life. They stated that after facing these limitations, they enjoyed the support, encouragement, and help of colleagues, friends, and others to overcome these limitations. Some participants explained that because talking to men about female genitals was taboo, they tried hard to hide the type of operation in their work environment. They stated that when co-workers realized the difficulty of moving and doing work, they helped a lot in doing hard work without asking why. Also, some participants described their experience of being encouraged by colleagues and workplace officials to pursue treatment. One participant said:

“After the hysterectomy, I couldn't do hard work. Since most of my colleagues were men, I could not talk to them about my surgery. But they helped me. They were careful that I do not get pressured while doing work.” (P6)

Many participants were concerned about limited information on hysterectomy and its complications. In this regard, they communicated with women who had previously had a hysterectomy. They became a source of information support for the participants. One participant said:

“I faced a lot of problems after my hysterectomy. I met several hysterectomized women. They guided me. I still get help from them.” (P8)

Some participants introduced their friends as a source of support. They said their friends gave them emotional support and care after the hysterectomy. A group of women stated that they took refuge in their friends and spent more time with them. One participant said:

“I hid my Surgery from my family, only my friend came with me. She took care of me.” (P13)

#### Supportive health providers

A group of health care providers and several physicians provided emotional support to participants during treatment and follow-up visits. During care for post-hysterectomy, receiving clinical care with expressions of affection from health personnel, including pain relief, nutritional assistance, and mobility, were some of the items described by participants. Some participants experienced severe pain. They said they had no hope of recovery at the time, but the information and guidance provided by the nurses were promising and alleviated their suffering. Some of the participants experienced fear combined with the feeling of loneliness in the recovery room. The fear and do not being aware of what had happened caused a feeling of insecurity in some participants in the early hours after the operation. The affection behavior of the doctor and the nurse made them feel safe. A group of participants described receiving emotional support and encouragement from health personnel. They stated that the support was beyond the physiological care expected of a physician or nurse during an illness. Some said that the kind behavior of the doctor and the health staff at the hospital motivated them to cope with the situation. One participant said:

“After hysterectomy, I was hospitalized in the ICU. That atmosphere was scary for me. But the ICU staff was excellent. I will not forget the energy I took from them in those days.” (P7)

## Discussion

This study explored the women's experiences of interdependency adaptation after hysterectomy under the guidance of the interdependence mode of the RAM. In the Roy Adaptation Model, two specific close relationships are explained in the mode of interdependence. The first explained relationship is the relationship with the important people who are very important to the person's life. The second is the relationship with support systems that point to those who help meet the needs of interdependence [22].

The results of this study showed that after hysterectomy, the interdependence between women and family members, including spouse, parents and children,, as well as the support system including family members, colleagues, friends, peers, medical staff increased. Participants experienced changes in their relationships with people who were important in their lives due to loneliness, changes in self-concept, loss of fertility. These changes ranged from increasing relationships to breaking up of relationships. Most of the changes described were related to changes in relationships with spouses and children. After gynecological surgeries, women found themselves different from others, and this difference was the result of a person’s interaction with herself and others [31]. The emotional breaking-up between couples due to decreased sexual desire and loss of fertility is one of the most important concerns of women who have undergone surgeries that cause menopause [32]. After a hysterectomy, women feel lonely and isolated. Feelings of shame, regret, anxiety, depression, and social isolation are some of the problems that affect women's social and family relationships after hysterectomy. Concerns about the husband's negative reactions to a hysterectomy caused women to distance themselves from their husbands to solve the problem, run away from the relationship with the husband, and sometimes allow the husband to remarry [33]. On the other hand, the results of this study showed an improvement in the relationship of many participants with their spouses. After evaluating their lives after a hysterectomy, women achieved positive aspects such as a chance to live again and enjoy with family especially spouses [34]. In this study, Most of the women improved their spiritual relationship after hysterectomy. They increased their spiritual relationship. They attended Quran recitation sessions and used prayer to bring peace. Spiritual communication, such as praying, reciting the Holy Quran, listening to music, walking, and sports (yoga), is one of the adaptive strategies used by people after a hysterectomy [4].

Loss of fertility after a hysterectomy increased women's emotional dependence on their children. Therefore, they became more sensitive to the care of their children. In some studies, women described fertility loss after hysterectomy as a loss of hopes and dreams [4]. In particular, women who underwent hysterectomy and experienced secondary infertility after hysterectomy faced whit a variety of issues, including defects in their integrity as a perfect woman which is often overlooked by health care providers[35]. Women with secondary infertility consider their current child to be a blessing from God. The limited number of children is stressful for these mothers. She and her family had high hopes for their existing child/children, leading to high levels of fear and stress [36].

In this study, participants described the association with an enhanced support system involving colleagues, friends, and the caregiver team, which was helpful for better adaptation to hysterectomy. However, in the literature review, no study was found that examined the role of the support system in adapting to hysterectomy. The need for compassionate behavior in care sectors and the organization of support groups have been identified for hysterectomies women in studies [32, 37]. Women's interaction with the community around them, receiving stimulus support, and encouraging support from family and friends are important strategies for overcoming the barriers and limitations of illness [38]. One source of support for women after a hysterectomy is their colleagues. The employed women who underwent hysterectomy were less anxious. One of the reasons was their involvement in society. Therefore, access to social support could be a strategy for adapting to hysterectomy [39].

Most participants supported by their family members. The support that they received included care, expressions of love, respect, and emotional support that helped women recover from the physical, psychological, and emotional consequences of the hysterectomy during their recovery period. This support gave participants a sense of calm and confidence. They also expressed mutual love, respect, and care for family members, friends, and relatives. These findings were confirmed by other studies [11, 37, 38]. In these studies, social support dimensions identified as emotional relationship, affectionate, positive social interaction, help, and care. After losing an organ of the body, women can better adapt to their social environment through the support, encouragement, and help of family, friends, or even a support group. In fact, these supportive behaviors are a way to overcome the obstacles and limitations of living with the loss of an organ [31]. According to the findings of this study, Husbands, as the most important source of support, play a significant role in women's adaptation to the hysterectomy. The provision of support and care of a husband and the accompaniment of a woman with a sexual partner represent a strong relationship that can not only reduce the stress of an important gynecological surgery such as a hysterectomy but also lead to closer relationships [40]. According to the findings of this study, women's interdependence after hysterectomy with family members and relatives is very important in adapting women to the hysterectomy. Husbands, as the most important source of emotional and care support, also play a significant role in women's adaptation to the Psychological aspects of hysterectomy. In Iranian society, especially at the time of encountering important events such as hysterectomy, family members play an important role in the processes of decision-making and care for the individuals. It can play a significant role in adapting women to hysterectomy.

This study is the first study to investigate the interdependence adaptation of women after hysterectomy under the guidance of RAM, which is a novelty aspect of this study. The findings of the present study explained the adaptation of women in the field of interdependence after hysterectomy, which can expand the existing knowledge in the field of compatibility with a hysterectomy and gynecological surgeries.

This study has two major limitations. First, this study was based on the experience of participants who wanted to share their experience after hysterectomy, so there may be other experiences that we have not been able to collect. Second, we considered only the experiences of women who underwent a hysterectomy. The views of husbands and other family members involved in women's relationships were not considered. This research has been conducted in an environment with different social and cultural structures due to the nature of qualitative studies. The number of participants is small and was selected by the purposeful sampling method. Therefore, generalizing the results may not be easy. In this regard, researchers tried to compensate for this limitation by moving based on strict observance of the principles of qualitative research and accurate reporting of research steps and efforts.

## Conclusion

The results of this study provide important information about post-hysterectomy adaptive behaviors in the field of interdependence to family members as important people in women's life and health care providers and other people as support systems. Based on this, nursing activities can be planned, and finally, the effectiveness of these activities can be evaluated. The findings of this study showed that women after hysterectomy feel a strong need to receive support from family members, health care providers, and colleagues. Therefore, it is recommended that a study be conducted with the presence of the husband, other family members, and health care providers who are involved in women's socio-emotional relationships and explore the views them.

## Data Availability

The datasets used and/or analyzed during the current study available from the corresponding author on reasonable request.
